# Finding Dr Florence Buchanan: The recovered face and fuller life story of a scientific legend

**DOI:** 10.1113/EP094202

**Published:** 2026-08-08

**Authors:** Brian C. Clark, Damian M. Bailey, Frances M. Ashcroft

**Affiliations:** ^1^ Ohio Musculoskeletal and Neurological Institute and Department of Biomedical Sciences Ohio University Heritage College of Osteopathic Medicine Athens Ohio USA; ^2^ Neurovascular Research Laboratory University of South Wales Pontypridd UK; ^3^ Department of Physiology, Anatomy and Genetics University of Oxford Oxford UK

**Keywords:** cardiac physiology, exercise physiology, history of physiology, reflexes, skeletal muscle, women in science

## Abstract

Dr Florence Buchanan (1867–1931) was one of physiology's foundational women. Trained as a zoologist at University College London (UCL), she became an exacting physiologist whose studies of muscle bioelectricity, reflexes, cardiac rhythm and exercise helped shape twentieth‐century physiology. She was the first woman to attend a meeting of The Physiological Society, one of its first six women members, a collaborator of Nobel laureates Charles Sherrington and August Krogh, and author of the first paper in *The Quarterly Journal of Experimental Physiology*, now *Experimental Physiology*. A Physiological Society ‘Blue Plaque’ at Oxford commemorates her. Yet although her papers and institutional importance survived, no authenticated photograph was known. We report the discovery of a captioned portrait in a 1941 Buchanan family memoir held by the National Library of Scotland. The memoir restores Dr Buchanan to view as daughter, sister, aunt, friend, teacher, cyclist, colleague and scientist despite her progressive visual impairment. Most strikingly, it records that she dictated her 1901 paper on the electrical response of muscle during three months of enforced bed rest for severe eye disease. We describe the portrait's provenance and memoir evidence, and consider an 1887 UCL practical‐zoology classroom photograph with Ray Lankester documenting her training environment. Because she studied there during this period, she may be among the three women students pictured, but no individual can be identified as her. The discovery restores not only a face but scale, texture and presence to a scientist whose work endured more visibly than our knowledge of the woman herself.

## Abstract (highlights)

- **What is the topic of this review?**
  This review reports the discovery of an authenticated photograph of Dr Florence Buchanan (1867–1931), the pioneering physiologist whose landmark 1908 paper opened the inaugural volume of *The Quarterly Journal of Experimental Physiology*, the predecessor to *Experimental Physiology*.
- **What advances does it highlight?**
  A securely captioned portrait in a 1941 family memoir held by the National Library of Scotland restores Dr Buchanan's visual identity and reveals new details of her humour, independence, friendships, devotion to children and students, and resilience despite progressive visual loss.

## 1 A PROMINENT SCIENTIST, ABSENT FROM THE VISUAL RECORD

Dr Florence Buchanan should not need rediscovery. By any reasonable historical measure, she was one of the foundational women of physiology. Born in 1867 into a scientifically distinguished family, she trained in zoology at University College London (UCL) and later moved into physiology at Oxford. Buchanan studied a remarkably broad range of subjects, including marine invertebrate morphology, muscle bioelectricity, reflex transmission, cardiac rhythm, pulse frequency, exercise and comparative physiology (Ashcroft, 2020; Bailey, 2026; Buchanan, 1889, 1893, 1894, 1899, 1901, 1908, 1909, 1912; Burgess, 2015; Clark, 2025; Krogh & Lindhard, 1913; Obituary, 1931; Smith, 1941; Tansey, 2015). Her work was meticulous, technically inventive and wide‐ranging.

Her career also intersected with several landmarks in the institutional history of physiology. She is believed to have been the first woman to attend a meeting of The Physiological Society (Ashcroft, 2020; Smith, 1941). When women were finally admitted to membership in 1915, she was among the first six elected and was listed first alphabetically (Burgess, 2015; Tansey, 2015). She worked alongside some of the most influential physiologists of the age, including Charles Sherrington (1857–1952) and August Krogh (1874–1949), both later Nobel laureates (Ashcroft, 2020; Clark, 2025; Krogh & Lindhard, 1913). Most fittingly for this journal, Dr Buchanan's 1908 paper, ‘On the time taken in transmission of reflex impulses in the spinal cord of the frog’, opened the first volume of *The Quarterly Journal of Experimental Physiology*, the direct predecessor of *Experimental Physiology* (Buchanan, 1908). More recently, The Physiological Society recognized her scientific legacy with a prestigious ‘Blue Plaque’ at the University of Oxford (Clark, 2025).

Despite these achievements, an important gap remained in the historical record. Her papers could be read, her scientific contributions assessed, her institutional barriers documented, and her place in the history of physiology increasingly restored (Ashcroft, 2020; Bailey, 2026; Burgess, 2015; Clark, 2025; Tansey, 2015). However, no authenticated photograph of Dr Buchanan was known, and the surviving record revealed comparatively little about her life beyond science (Ashcroft, 2020; Bailey, 2026; Clark, 2025). Recent accounts rightly presented Dr Buchanan as pioneering, exacting, technically capable and overlooked, but these qualities alone could not fully recover the person behind the work. Why her visual record is so sparse is unclear: it may be because she disliked being photographed or lacked time to sit for a portrait, but no source reviewed here documents such a preference; the absence of surviving images cannot itself establish why her visual record is so sparse. The newly identified material now allows that professional profile to be complemented by a more personal account of the woman behind the work.

The absence of any authenticated photograph or portrait of Dr Buchanan was sufficiently troubling that it prompted one of us to create an AI‐assisted, evidence‐informed documentary portrait of how she might have appeared c. 1907–1909 (Bailey, 2026). The image was generated iteratively from tightly constrained textual evidence – including Dr Buchanan's published scientific corpus, contemporary accounts of her working practices and personal recollections from those who knew her – and was presented explicitly as a historically plausible visual hypothesis, not a definitive likeness.

For the present review, ChatGPT 5.5 Pro was used more narrowly to broaden search terms and identify possible archival leads. It did not authenticate or analyse the recovered portrait. Authentication depended on conventional archival evidence: the portrait's caption, its placement opposite Smith's remembrance of Dr Buchanan, the volume's list of illustrations and the National Library of Scotland's archival record. The portrait could in principle have been found through conventional searching; AI served only as a search aid.

## 2 FINDING FLORENCE IN THE FAMILY ARCHIVE

**FIGURE 1:**
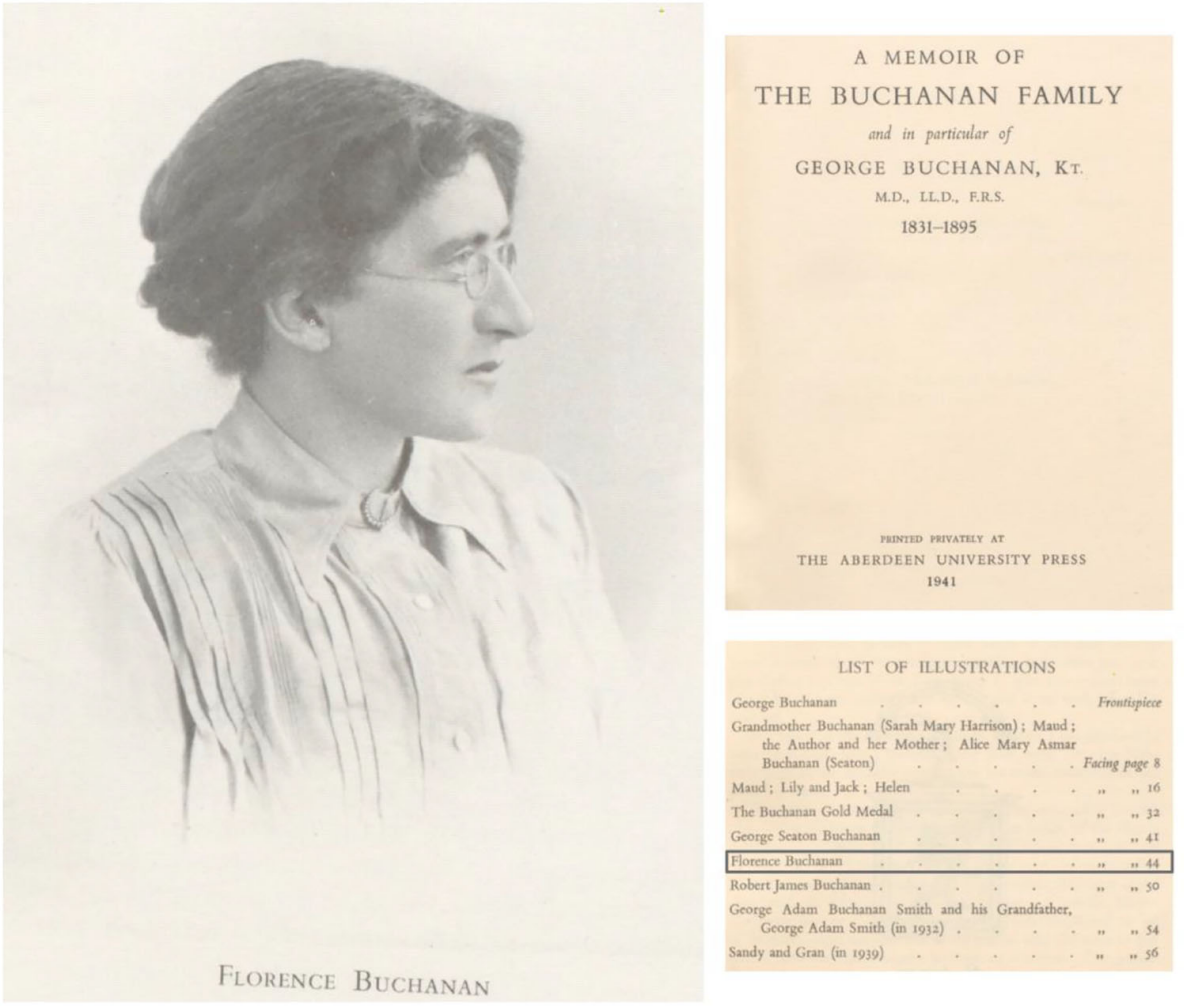
Florence Buchanan, restored to view. Left, an undated photographic portrait captioned ‘Florence Buchanan’ in Lilian Adam Smith's privately printed 1941 Buchanan family memoir. Upper right, the title page of *A Memoir of the Buchanan Family, and in particular of George Buchanan, Kt., M.D., LL.D., F.R.S., 1831–1895*, printed privately at the Aberdeen University Press in 1941. Lower right, the list of illustrations, which identifies ‘Florence Buchanan’ as the plate facing page 44. *Source: A Memoir of the Buchanan Family*, National Library of Scotland, Acc.9446/400. Digital images supplied by The National Library of Scotland, which does not claim copyright in digital images it creates of its archive collections and does not require a licence or fee for their reuse. The photographer of the original portrait is unknown. Credit: The National Library of Scotland.

The portrait did not emerge from a physiology archive filed under Dr Buchanan's name. It was found in a book about her father (Smith, 1941). The source is a privately printed 1941 volume, *A Memoir of the Buchanan Family, and in particular of George Buchanan, Kt., M.D., LL.D., F.R.S., 1831–1895*, held by the National Library of Scotland as Acc.9446/400 (Smith, 1941). The book was written by Dr Buchanan's sister, Lilian Adam Smith, and centres on their father, Sir George Buchanan, the distinguished public‐health physician. Within it, however, is a section headed ‘Florence Buchanan, D.Sc. (1867–1931)’. Opposite that remembrance is an undated photographic portrait of a bespectacled woman shown in profile. Beneath the image are the words ‘Florence Buchanan’ (Figure 1).

The identification is clear from the volume itself. The title page identifies the book as a privately printed Buchanan family memoir issued by Aberdeen University Press in 1941. Its list of illustrations names ‘Florence Buchanan’ as the plate facing page 44; the adjacent text is a remembrance devoted to her, and the portrait itself is captioned with her name. The photographer and date of the original image are not yet known, but Dr Buchanan's identity is not in doubt. The photograph comes from a named family source, created only a decade after Dr Buchanan's death, and preserved in the National Library of Scotland.

The place where the photograph was found is telling. Florence had not disappeared because the evidence was lost; she was difficult to find because the evidence was filed within a family memoir organized around her father rather than within the formal record of physiology. Dr Buchanan appears within it as daughter, sister and family member, not as physiologist, experimentalist or scientific pioneer. A search limited to ‘women physiologists’, ‘Oxford physiology’ or even ‘Florence Buchanan’ could easily miss her. The problem was not that the evidence had disappeared. It was that the route to it passed through the family history of a famous man.

This pattern is familiar in histories of women in science. Published achievements may remain visible while personal lives, relationships and recognition recede, particularly when relevant evidence survives in domestic rather than institutional collections (Abir‐Am & Outram, 1987; Rossiter, 1993). Faces, letters and remembered habits may persist in family volumes, private albums, memoirs, uncatalogued photographs and archival margins. In Dr Buchanan's case, the missing image was not in the formal apparatus of physiology. It was waiting in the family record.

These sources do not carry equal evidentiary weight. The memoir's internal captioning and placement provide strong provenance for the portrait, but its biographical recollections are retrospective family testimony, written by an affectionate sister approximately a decade after Dr Buchanan's death. Like any commemorative memoir, it is selective and shaped by memory, family loyalty and the volume's purpose. We therefore treat its anecdotes and character assessments as Smith's testimony rather than independently verified fact, and distinguish them from claims corroborated by publications, institutional records or other sources. This limitation does not diminish the memoir's value; it defines the kind of evidence it provides.

## 3 A FATHER, A DAUGHTER AND A SCIENTIFIC LIKENESS

**FIGURE 2:**
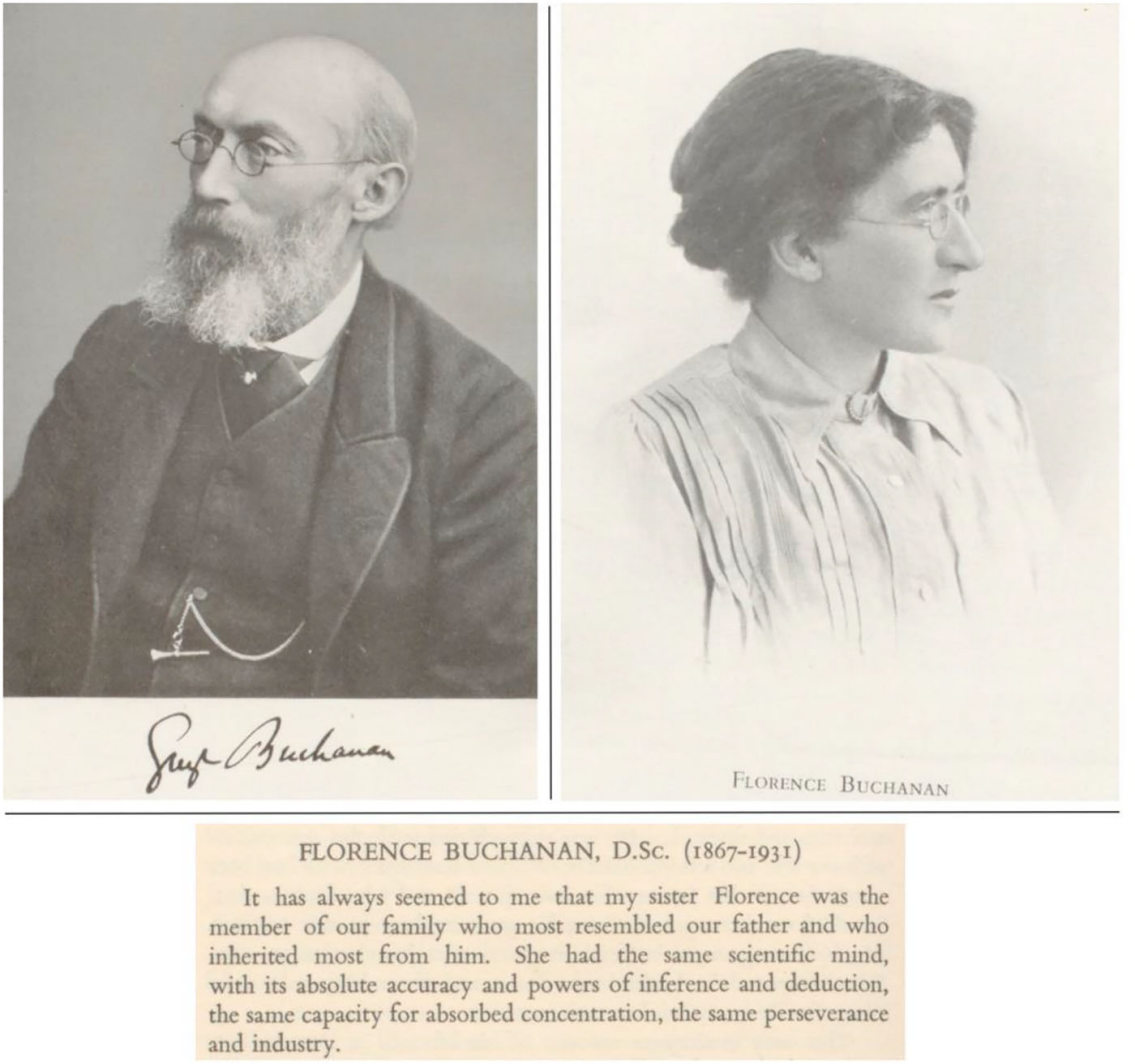
Family likeness and family testimony. Left, Sir George Buchanan, reproduced as the frontispiece to Lilian Adam Smith's privately printed 1941 Buchanan family memoir. Right, Florence Buchanan, from the portrait captioned ‘Florence Buchanan’ facing page 44. Bottom, the opening of Smith's remembrance, in which she writes that Florence was the family member who ‘most resembled our father’ and inherited his scientific mind, accuracy, concentration, perseverance and industry. The juxtaposition illustrates both the family resemblance and the way Buchanan was remembered within her family: not only as Sir George's daughter, but as the relative who most fully carried forward his habits of mind. Source: *A Memoir of the Buchanan Family*, National Library of Scotland, Acc.9446/400. Digital images supplied by The National Library of Scotland, which does not claim copyright in digital images it creates of its archive collections and does not require a licence or fee for their reuse. The photographers of the original portraits are unknown. Credit: The National Library of Scotland.

The memoir does more than identify Dr Buchanan's portrait. It also begins its remembrance by linking her closely to her father. Lilian Adam Smith wrote that Florence was the family member who most resembled Sir George Buchanan and who inherited the most from him: a scientific mind, accuracy, concentration, perseverance and industry (Smith, 1941). The memoir's frontispiece shows Sir George in profile; the newly recovered portrait shows Dr Buchanan in a similar orientation (Figure 2). Placed side by side, the resemblance is striking, particularly in the forehead, profile and nose; the shared profile orientation and wire‐rimmed spectacles also invite comparison.

The more important point is biographical. Her sister did not present Dr Florence Buchanan simply as the daughter of an eminent father. She presented her as the family member who most fully embodied his intellectual habits. The resemblance was not merely facial but temperamental: precision, concentration, perseverance and industry. These qualities are evident throughout Dr Buchanan's published science, from the careful morphological descriptions of her early zoological studies to the delicate recording, instrumental adaptation and disciplined interpretation required in her later physiology. The family therefore recognised in Florence not simply a likeness, but a shared scientific method.

This point also helps guard against a familiar distortion. Dr Buchanan's story could easily be reduced to that of a woman working in the shadow of famous men: her father, Burdon Sanderson, Sherrington, Krogh and the male‐dominated academic institutions of the time. The memoir places her within that world, but it also makes clear that she was not defined by it. Family and institutions helped shape her path, but her scientific identity was her own. The qualities she shared with Sir George became, in her hands, the foundation of an independent scientific career.

## 4 AN 1887 PHOTOGRAPH OF DR BUCHANAN'S UCL TRAINING ENVIRONMENT

**FIGURE 3:**
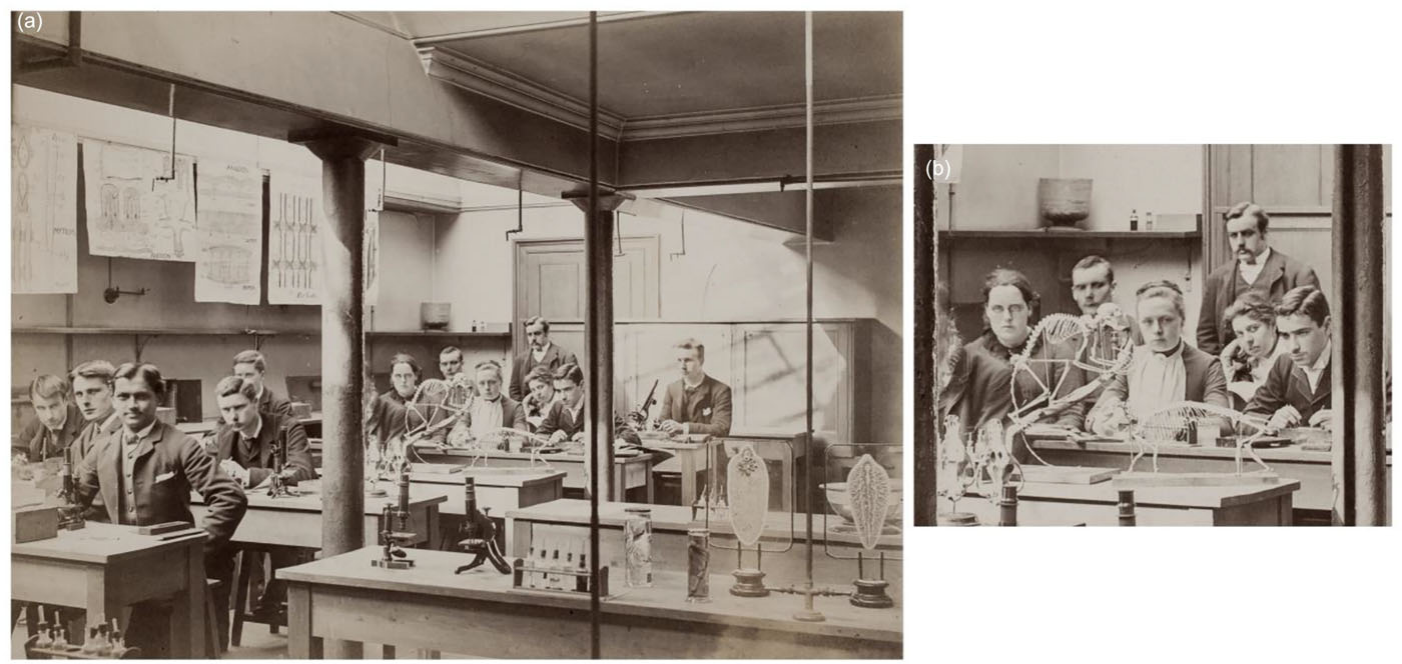
E. Ray Lankester's 1887 ‘Longer Course in Practical Zoology’ at University College London. (a) Full classroom photograph. (b) Enlarged view of the central group, including three women. Dr Florence Buchanan studied zoology at UCL under Lankester from 1886 to 1890 and may be among those pictured, but no annotated print, name key or course register has been found that identifies the individuals. The left‐hand woman appears to be wearing small round spectacles, but this feature is not diagnostic, and the available historical and visual evidence cannot establish whether Dr Buchanan is present or, if she is, which woman she is. The photograph therefore securely documents the laboratory environment in which Dr Buchanan trained but does not provide an authenticated image of her. The image was cropped and enlarged for display; no generative enhancement was used. Classroom photograph source: UCL Museums & Collections/UCL Archives, UCLCA/7/2/3/66. UCL Museums & Collections dates the classroom series to 1887 (Davidson, 2016). The photographer is unknown; no reproduction permission is required because of the image's age.

The recovered portrait also invites assessment of another image. UCL preserves a photograph of students in E. Ray Lankester's ‘Longer Course in Practical Zoology’ (Figure 3). UCL's archival metadata place it in the 1880s, while the UCL Museums & Collections website dates this classroom series specifically to 1887 (Davidson, 2016). The room is densely alive with late Victorian science: benches crowded with microscopes and glassware, charts on the walls, large teaching models rising above the tables, and students gathered around the apparatus of practical zoology. Three women are visible in the central rear group.

Dr Buchanan studied zoology at UCL under Lankester from 1886 to 1890 and would have been approximately 20 years old in 1887 (Ashcroft, 2020; Clark, 2025; Obituary, 1931; Smith, 1941). It is therefore possible that she is among the three women students pictured. The archival scan permits closer examination of the three women in the central group. The left‐hand woman appears to be wearing small round spectacles, but this feature is not diagnostic. Neither visual comparison with the later authenticated portrait nor the available historical evidence can establish whether Dr Buchanan is present or, if she is, which woman she is. Other women studied science at UCL during this period, and no annotated print, name key, course register or other independent documentation has been found that identifies those pictured.

The photograph should therefore be understood primarily as a record of Dr Buchanan's educational environment rather than as an identified portrait. It documents a working practical‐zoology classroom in which women were a small minority. Although Dr Buchanan may be among the students pictured, independent corroborating evidence would be required to establish her presence or identify her within the group.

## 5 FROM ZOOLOGY TO THE OPENING PAPER OF THIS JOURNAL'S PREDECESSOR

Dr Buchanan entered research through zoology. At UCL she trained under Lankester and developed expertise in morphology and marine invertebrates. Her early publications on polychaete worms and a branchiate polynoid show a young scientist already committed to careful observation and precise description (Buchanan, 1893, 1894). Although the subject was zoological, the experimental discipline would carry into her later physiology.

By the mid‐1890s she had moved into physiology at Oxford, working in the laboratory of John Burdon Sanderson (Clark, 2025). Marine invertebrates gave way to muscle, nerve and heart, but her scientific approach was consistent: attentive to structure, alert to function, and willing to adapt instruments to make biological processes visible. She investigated the electrical response of muscle, the timing of reflex transmission, the rate and form of the heartbeat, and the physiological significance of pulse frequency across species and conditions (Buchanan, 1899, 1901, 1908, 1909, 1912). She recorded the hearts of small mammals, birds, reptiles and human volunteers. She investigated exercise, hibernation and the relationship between physiological state and measurable rhythm.

This was not ornamental participation in another scientist's programme. Dr Buchanan was an accomplished experimentalist in her own right. She built, adapted and used apparatus (Ashcroft, 2020; Clark, 2025). She published substantial papers. She asked quantitative questions at a time when physiology was becoming increasingly instrumented and integrative (Ashcroft, 2020; Clark, 2025). Her work sat at the intersection of observation, technology and mechanistic interpretation.

## 6 SIXTY‐SIX PAGES DICTATED FROM BED

The most arresting new passage in the family memoir concerns Dr Buchanan's 1901 paper, ‘The electrical response of muscle in different kinds of persistent contraction’ (Buchanan, 1901). During this period her eyesight deteriorated so severely that she was ordered to lie flat on her back for three months (Smith, 1941). Unable to write, she dictated the paper to a friend (Smith, 1941). Smith records that the friend was astonished by the accuracy of Dr Buchanan's memory (Smith, 1941).

The detail is extraordinary. The published paper spans 66 pages and addresses technically demanding questions in muscle electrophysiology. To dictate such a work from bed required more than persistence. Buchanan had to hold in memory the structure of an extended scientific argument: methods, observations, interpretations, terminology, literature and sequence. She had to compose without the usual visual and physical routines of writing and revision. The memoir also notes that the University of London subsequently awarded her a D.Sc., largely in recognition of this work (Smith, 1941).

This episode should not be reduced to a sentimental triumph over adversity. Its deeper significance lies in what it reveals about scientific authorship under constraint. Dr Buchanan retained intellectual command, but the work was made possible through collaboration, care and adaptation. A friend supplied the hand she could not use. Later, students and assistants helped with recordings and practical arrangements (Smith, 1941). The science was unmistakably Dr Buchanan's, but its production depended on a social infrastructure of collaboration, care and practical support as much as on intellectual command.

The episode also sharpens our understanding of her later career. Dr Buchanan's visual impairment was not an incidental biographical detail. It shaped the way she worked, moved, recorded, remembered and communicated. The newly recovered memoir therefore reveals not only the finished paper, but the demanding personal and social circumstances from which it emerged.

## 7 THE FULLER PERSON IN THE FAMILY RECORD

Scientific biography often compresses a life into a few durable traits. Dr Buchanan has usually been remembered, when she has been remembered at all, as exacting, technically gifted, determined and professionally constrained by the male institutions around her. Smith's memoir does not overturn that portrait; it offers a sister's more intimate and necessarily selective perspective, giving it depth, warmth and human scale.

Lilian Adam Smith knew this was necessary. ‘No record of Florence would be right,’ she wrote, ‘that did not tell of her love of children and young people’ (Smith, 1941). It is a striking sentence because it shifts the frame immediately. The formidable physiologist was also ‘Aunt Florence’, a woman who, Smith recalled, kept her ‘young and loving heart’ to the end of her life (Smith, 1941). Children were drawn to her (Smith, 1941). They watched her make paper birds and frogs, boats and boxes, and cut strings of small figures (Smith, 1941). She listened to their stories and remembered their sayings long after others had forgotten them (Smith, 1941). Nieces travelling from Cheltenham to Oxford were not treated as interruptions to serious work; they were welcomed into a world of river picnics, strawberry teas, country excursions, open‐air theatre and college visits (Smith, 1941). A half‐term holiday with Aunt Florence, Smith wrote, was ‘a treat eagerly looked forward to’ (Smith, 1941).

Her scientific life was also more social than the formal record suggests. Among Oxford undergraduates, men and women alike, she had ‘many devoted friends’, some of whom assisted her by allowing recordings of their heartbeats before and after exercise (Smith, 1941). Smith remembered young athletes who would ‘dash into her laboratory after a race’ so that Dr Buchanan could capture the physiological aftermath (Smith, 1941). The detail is vivid because it shows the laboratory not as an isolated room but as a place of shared curiosity and trust. ‘Her enthusiasm was contagious,’ Smith wrote, and through it many others came to recognize ‘the fascination and interest of scientific investigation’ (Smith, 1941).

These recollections do not soften the seriousness of Dr Buchanan's science; they explain part of how it worked. Her experiments drew on relationships – with students, athletes, colleagues, assistants and friends – who helped make possible the measurements she pursued. Nor was her interest in young people confined to physiology. She was fascinated by airmen, whose work thrilled her with its ‘accuracy and daring’ (Smith, 1941). During the First World War she made friends among soldiers in the Oxford hospitals and mourned deeply, and proudly, when two of her nephews were killed in action (Smith, 1941).

The memoir also protects Dr Buchanan from caricature. Later recollections of eccentricity or indifference to appearance may contain truth, especially for her later years, but they are not enough (Mitchison & Squier, 1995). Smith's account presents a woman physically limited yet adventurous, private in some respects yet deeply connected, exacting yet playful (Smith, 1941). She was not simply withdrawn into experiment. She brought people toward it.

One anecdote captures this mixture of vulnerability, humour and defiance. Long after her sight had nearly gone, Dr Buchanan continued to ride a bicycle, ‘to the agitation and alarm of all who saw her’. When a relative urged her to stop, she answered: ‘Do not ask me to give up the one bit of adventure that is left in my life’ (Smith, 1941). The line is comic, stubborn and poignant at once. It is also entirely consistent with her scientific character: a refusal to surrender more ground than necessity demanded.

## 8 WORKING AS SIGHT FAILED

Dr Buchanan's progressive visual impairment became one of the defining conditions of her later life. The memoir attributes her loss of sight to retinal detachment (Smith, 1941). It describes pain from strong light, increasing blindness and a working room kept so unchanged that she could navigate it by memory and touch (Smith, 1941). Visitors sometimes underestimated how little she could see because her hands moved with such assurance.

She nevertheless continued to work. In that darkened laboratory, she handled materials by touch, explained apparatus she had designed or adapted, and used a typewriter and specially sized paper when ordinary writing became impossible (Smith, 1941). The laboratory became an extended memory system: room, bench, instruments, body and mind arranged so that scientific work could continue despite diminishing sight (Smith, 1941).

Even her visual symptoms became scientific material. Dr Buchanan recorded the shapes, colours and effects produced by her retinal disorder in the hope that her experience might help others (Smith, 1941). She ultimately bequeathed her eyes for scientific study (Smith, 1941). This was not simply a story of ‘overcoming blindness.’ It was the reorganization of scientific practice around changing physical capacities. Dr Buchanan did not cease being an observer when sight failed; she changed how observation could be performed.

That point matters for how we remember her. Disability historians have cautioned against framing disabled lives only as tragedy or heroic overcoming (Kudlick, 2003). Dr Buchanan's life resists both simplifications. Her impairment closed some routes and made others painful or difficult. It also produced new forms of adaptation, collaboration and self‐observation. She continued to work not because disability was irrelevant, but because she found ways to make science possible within it.

## 9 MYSTERIOUS NIGHT

During her final illness in 1931, Buchanan ‘frequently repeated’ Joseph Blanco White's sonnet ‘Mysterious Night’, which her sister described as beloved by their father (Smith, 1941). Smith then wrote that Buchanan ‘passed from her darkness to the light’ and reproduced the poem in full at the close of her remembrance. Its inclusion therefore has a direct biographical basis: it records words to which Buchanan returned near death and connects her final illness with the memory of her father.

Mysterious Night! when our first parent knew

Thee from report divine, and heard thy name,

Did he not tremble for this lovely frame,

This glorious canopy of light and blue?

Yet, ’neath a curtain of translucent dew,

Bathed in the rays of the great setting flame,

Hesperus with the host of heaven came,

And lo! Creation widened in man's view.

Who could have thought such darkness lay concealed

Within thy beams, O Sun! or who could find

Whilst flower and leaf and insect stood revealed,

That to such countless orbs thou mad'st us blind!

Why do we then shun Death with anxious strife?

If Light can thus deceive, wherefore not Life?

—Joseph Blanco White (1775–1841)

The poem's imagery has clear points of contact with Dr Buchanan's experience of progressive blindness. She worked in a darkened room, navigated her laboratory by memory and touch, and recorded the visual phenomena associated with her retinal detachment in the hope that they might help others. Beyond these documented associations, however, any more precise interpretation of what the poem meant to Dr Buchanan would necessarily be speculative.

## 10 SEEING DR FLORENCE BUCHANAN MORE CLEARLY

The rediscovered portrait does not change Dr Buchanan's scientific legacy, but it gives us a more complete understanding of the person behind that legacy. We can now see the mature scientist her family preserved. The UCL classroom photograph also allows us to see the environment in which a younger Dr Buchanan trained; she may be among the students pictured, but the photograph does not establish this. We can connect the first author of this journal's predecessor to a life marked by precision, visual impairment, friendship, determination, care and adventure. The papers can finally be viewed alongside the person.

Historical biography often begins with what is missing. In Dr Buchanan's case, the missing portrait has now been found, and with it a fuller account of the person behind the science. *Experimental Physiology* can now look back with greater accuracy at the woman who opened its published record. More than that, it can now see her.

## AUTHOR CONTRIBUTIONS

Brian C. Clark: Conceptualization; investigation; archival research; visualization; writing – original draft; writing – review and editing. Damian M. Bailey: Conceptualization; investigation; writing – review and editing. Frances M. Ashcroft: Conceptualization; investigation; writing – review and editing. All authors have read and approved the final version of this manuscript and agree to be accountable for all aspects of the work in ensuring that questions related to the accuracy or integrity of any part of the work are appropriately investigated and resolved. All persons designated as authors qualify for authorship, and all those who qualify for authorship are listed.

## CONFLICT OF INTEREST

D.M.B. is Editor‐in‐Chief of Experimental Physiology.

## GENERATIVE AI STATEMENT

During the preparation and revision of this manuscript, the authors used ChatGPT (OpenAI) to assist with archival searching, identifying potential source leads, organizing and synthesizing source material, and drafting and editing portions of the text. ChatGPT was not used to authenticate the portrait or identify individuals in the UCL photograph. All AI‐assisted output was critically reviewed, revised and verified against original sources by the authors, who take full responsibility for the final content.

## References

[eph70417-bib-0001] Abir‐Am, P. G. , & Outram, D. (1987). Uneasy careers and intimate lives: Women in science 1789‐1979, Rutgers University Press.

[eph70417-bib-0002] Ashcroft, F. (2020). Florence Buchanan: A true pioneer. Physiology News, 127, 46–47.

[eph70417-bib-0003] Bailey, D. M. (2026). Against erasure: Restoring Dr Florence Buchanan's missing image in physiology. Experimental Physiology, 111(5), 2416–2422. 41731652 10.1113/EP093649 PMC13131103

[eph70417-bib-0004] Buchanan, F. (1889). On the ancestral development of the respiratory organs in the Decapodous Crustacea. Journal of Cell Science, S2‐29(116), 451–467.

[eph70417-bib-0005] Buchanan, F. (1893). Report on the polychaetes collected during the Royal Dublin Society's survey off the west coast of Ireland. Pt. 1. Deep water forms. Proceedings of the Royal Dublin Society (New Series), 8(2), 169–179.

[eph70417-bib-0006] Buchanan, F. (1894). A polynoid with branchiae (Eupolyodontes cornishii). Quarterly Journal of Microscopical Science, London, New Series, 35, 433–450.

[eph70417-bib-0007] Buchanan, F. (1899). The efficiency of the contraction of veratrinised muscle. The Journal of Physiology, 25(2), 137–156. 10.1113/jphysiol.1899.sp000783 PMC1516664 16992522

[eph70417-bib-0008] Buchanan, F. (1901). The electrical response of muscle in different kinds of persistent contraction. The Journal of Physiology, 27(1‐2), 95–160.15. 16992604 10.1113/jphysiol.1901.sp000864 PMC1540546

[eph70417-bib-0009] Buchanan, F. (1908). On the time taken in transmission of reflex impulses in the spinal cord of the frog. Quarterly Journal of Experimental Physiology, 1(1), 1–66.

[eph70417-bib-0010] Buchanan, F. (1909). The physiological significance of the pulse rate. Trans Oxford University Scientific Club, 34, 351–356.

[eph70417-bib-0011] Buchanan, F. (1912). Observations of the movements of the heart by means of electrocardiograms. Proceedings of the Royal Society of Medicine, 5, 185–187. 10.1177/003591571200500268 PMC2006054 19975651

[eph70417-bib-0012] Burgess, H. (2015). 100 years of women members: The Society's centenary of women's admission. Physiology News, 98, 34–35.

[eph70417-bib-0013] Clark, B. C. (2025). Blue plaque review series: Dr Florence Buchanan: A trailblazing physiologist in an era of barriers and breakthroughs. Experimental Physiology, 110(9), 1203–1216. 40259511 10.1113/EP092228 PMC12400829

[eph70417-bib-0014] Davidson, T. (2016). Specimen of the Week 244: The historic wax flatworm. UCL Museums & Collections Blog, https://blogs.ucl.ac.uk/museums/2016/06/17/specimen‐of‐the‐week‐244‐the‐parasitic‐flatworm‐wax‐flatworm/

[eph70417-bib-0015] Krogh, A. , & Lindhard, J. (1913). The regulation of respiration and circulation during the initial stages of muscular work. The Journal of Physiology, 47(1–2), 112–136. 16993229 10.1113/jphysiol.1913.sp001616 PMC1420474

[eph70417-bib-0016] Kudlick, C. J. (2003). Disability history: Why we need another “other”. American Historical Review, 108(3), 763–793. 12964564 10.1086/529597

[eph70417-bib-0017] Mitchison, N. A. , & Squier, S. A. (1995). Solution three. The Feminist Press at CUNY.

[eph70417-bib-0018] Obituary . (1931). Dr. Florence Buchanan. Nature, 127, 456.

[eph70417-bib-0019] Rossiter, M. W. (1993). The Matthew Matilda effect in science. Social Studies of Science, 23(2), 325–341.

[eph70417-bib-0020] Smith, L. A. (1941). *A memoir of the Buchanan family, and in particular of George Buchanan, Kt., M.D., LL.D., F.R.S*., 1831–1895. Aberdeen University Press.

[eph70417-bib-0021] Tansey, T. (2015). Women and the early *Journal of Physiology* . The Journal of Physiology, 593(2), 347–350. 25630254 10.1113/jphysiol.2014.288258 PMC4303378

